# High-Throughput Identification of Mammalian Secreted Proteins Using Species-Specific Scheme and Application to Human Proteome

**DOI:** 10.3390/molecules23061448

**Published:** 2018-06-14

**Authors:** Jian Zhang, Haiting Chai, Song Guo, Huaping Guo, Yanling Li

**Affiliations:** 1 School of Computer and Information Technology, Xinyang Normal University, Xinyang 464000, China; songguo_xynu@yeah.net (S.G.); hpguoxynu@sina.com (H.G.); yanlingli639@163.com (Y.L.); 2 College of Medical, Veterinary and Life Sciences, University of Glasgow, Glasgow G12 8QQ, UK; h.chai.1@research.gla.ac.uk

**Keywords:** secreted proteins, species-specific, high-throughput, human proteome

## Abstract

Secreted proteins are widely spread in living organisms and cells. Since secreted proteins are easy to be detected in body fluids, urine, and saliva in clinical diagnosis, they play important roles in biomarkers for disease diagnosis and vaccine production. In this study, we propose a novel predictor for accurate high-throughput identification of mammalian secreted proteins that is based on sequence-derived features. We combine the features of amino acid composition, sequence motifs, and physicochemical properties to encode collected proteins. Detailed feature analyses prove the effectiveness of the considered features. Based on the differences across various species of secreted proteins, we introduce the species-specific scheme, which is expected to further explore the intrinsic attributes of specific secreted proteins. Experiments on benchmark datasets prove the effectiveness of our proposed method. The test on independent testing dataset also promises a good generalization capability. When compared with the traditional universal model, we experimentally demonstrate that the species-specific scheme is capable of significantly improving the prediction performance. We use our method to make predictions on unreviewed human proteome, and find 272 potential secreted proteins with probabilities that are higher than 99%. A user-friendly web server, named iMSPs (identification of Mammalian Secreted Proteins), which implements our proposed method, is designed and is available for free for academic use at: http://www.inforstation.com/webservers/iMSP/.

## 1. Introduction

Secreted proteins (SPs) are the proteins that are released by a cell or tissue into the extracellular space. Generally, these proteins are produced through two pathways, namely the classical Endoplasmic Reticulum and Golgi routes [1] and the unclassical secretory routes [2]. Secreted proteins play important roles in living organisms. According to their functions, they could be divided into many categories, which include hormones [3], cytokines [4], enzymes [5], toxins [6], and antibiotics [5]. In humans, the liver is the most important secretory organ. It produces a large number of plasma proteins, such as albumin, fibrinogen, and transferrin. Secreted proteins are easy to detect in body fluids, urine, and saliva in clinical trials [7], which endows them with the capability of being a rich source of biomarkers and drug targets. Since the majority of the blood diagnostic tests are directly towards secreted proteins, it is not unusual to emphasize the significance of this class of proteins.

Recent years have witnessed a number of computation-based approaches in this field. In 2011, Hong et al. used physiochemical properties and amino acid composition features to predict whether a protein can be excreted into urine [8]. Liu et al. adopted an information-retrieval (i.e., manifold ranking) technique for the identification of blood-secretory proteins [9]. Huang et al. used 531 physicochemical properties together with support vector machine to recognize secreted proteins [10]. Soon after that, Restrepo-Montoya et al. calculated a set of sequence-based features to predict secreted proteins and proposed a classifier named NClassG+ [11]. Yu et al. predicted bacterial secreted proteins by using the general concept of pseudo amino acid composition [12]. They also constructed a public web server, named SecretP. In [13], Luo et al. combined position-specific scoring matrix and used auto-covariance theory to encode secreted proteins. In 2013, Wang et al. collected a series of physicochemical properties and several sequence-based features for identifying human salivary proteins from blood circulation. They also used their method in diagnostic biomarker recognition [14]. Yu et al. built a multi-classifier to predict various types of secreted proteins [15]. Besides simply predicting secreted proteins, Sun et al. applied their method in the identification of head and neck cancer biomarkers [16]. Additionally, in secreted proteins, signal peptides are destined towards the secretory pathway [17]. Therefore, the research on signal peptides contribute to the knowledge of secreted proteins [18,19,20]. However, proteins with signal peptides are not necessarily secreted [21]. In eukaryotes, a protein with signal pepteides will cotranslationally translocate across the membrane. While in prokaryotes, this process takes across the cytoplasmic membrane [21].

The above-mentioned studies all contributed to the development of the research in secreted proteins. However, there still exist some shortcomings that need to be further investigated: (i) the structure-based methods, which could achieve high accuracy, are limited in real application due to the small number of known protein structures. Although sequence-based predictors are featured in their wide application, they often suffer from the unsatisfactory prediction performance; (ii) many methods used structure- or sequence-based features in order to construct feature matrix without analyzing the features in detail. That is, it is unknown whether these features could successfully differentiate secreted proteins from non-secreted proteins; (iii) some predictors simply predict general secreted proteins without considering the differences across various species of secreted proteins. Based on our investigation, the differences do exist and they help to recognize specific secreted proteins.

In view of these three issues, we aim to focus on the challenge of proposing an accurate computational method for the identification of mammalian secreted proteins based on primary sequences. Instead of using general secreted proteins, we compile several sub-datasets of prevalent species of secreted proteins. The features which have been proved to be involved in secreted proteins are collected to encode secreted proteins. We analyze the differences between secreted proteins and non-secreted proteins in detail, especially across several mammalian species. In addition, Fisher-Markov selector together with incremental feature selection scheme is introduced to remove redundant features, as well as to explore optimal feature subset. Experimental results on benchmark datasets and independent testing dataset prove the effectiveness and generalization capability of our method. Additionally, we also make predictions on unreviewed human proteome and find potential secreted proteins with high confidence.

## 2. Results and Discussion

### 2.1. The Characteristics of the Calculated Features

**Figure 1:**
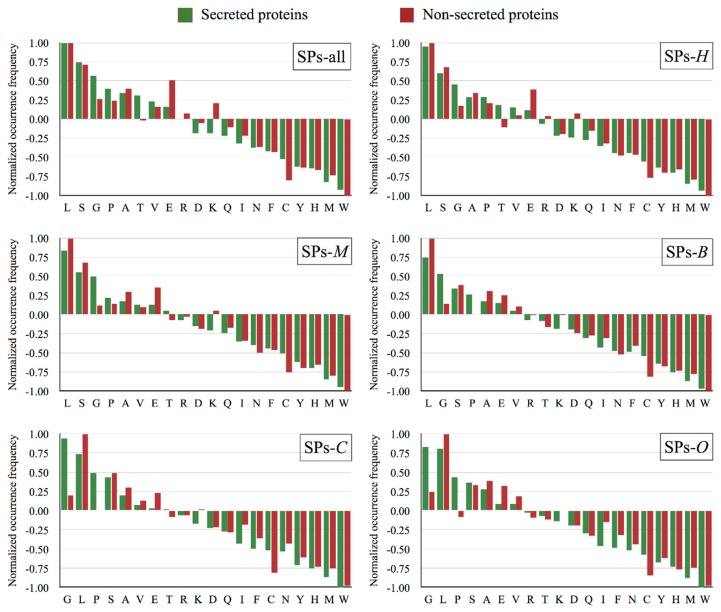
The relative amino acid composition of secreted proteins and non-secreted proteins in various datasets. The amino acids are sorted according to their enrichments in secreted proteins.

In this paper, we encode the proteins by using three types of features, including amino acid composition (AAC), sequence motifs (MTF), and physicochemical properties (PCP). Before constructing the prediction model, we investigate the differences of the considered features between secreted proteins and non-secreted proteins. As shown in Figure 1 (SPs-all), eight amino acids are overrepresented in secreted proteins against that in non-secreted proteins. When compared with non-secreted proteins, five out of eight show relative higher overrepresented. Lysine is less favored in secreted proteins when compared with that in non-secreted proteins. In five specific-specific datasets, the top five enriched amino acids keep consensus with that in SPs-all. However, the frequencies vary in different species. These results indicate that amino acids composition help to discriminate secreted proteins from non-secreted proteins.

**Figure 2:**
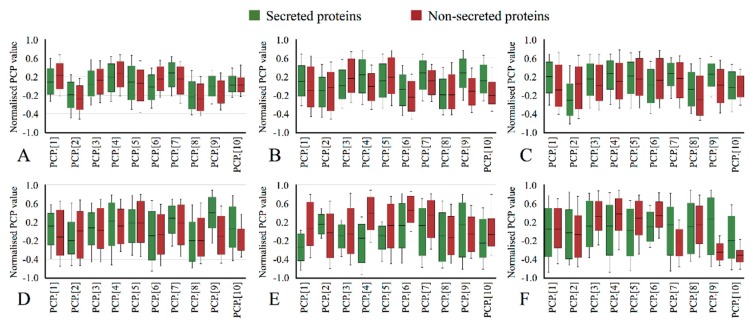
Physicochemical properties of secreted proteins and non-secreted proteins in (**A**) Secreted proteins (SPs)-all, (**B**) SPs-*H*, (**C**) SPs-*M*, (**D**) SPs-*B*, (**E**) SPs-*C* and (**F**) SPs-*O*. Physicochemical properties (PCP) represent hydrophobicity (PCP. [1]), polarity (PCP. [2]), solvation free energy (PCP. [3]), graph shape index (PCP. [4]), transfer free energy (PCP. [5]), correlation coefficient in regression analysis (PCP. [6]), residue accessible surface area (PCP. [7]), partition coefficient (PCP. [8]), entropy of formulation (PCP. [9]) and protein kinase A (PCP. [10]), respectively..

We further illustrate the distribution of physicochemical properties in Figure 2. Midline, box boundaries, and whiskers indicate median, quartiles, and 10th and 90th percentiles. The *x*-axis indicates the normalized values and *y*-axis stands for twelve properties. For instance, the distribution of secreted proteins against the non-secreted proteins varies obviously in hydrophobicity (Panel A). This phenomenon keeps consistent in SPs-*H*, SPs-*M*, SPs-*B*, and SPs-*C*. In SPs-*H* or SPs-*B*, a significant difference is found on the distributions of the entropy of formulation and protein kinase A. In SPs-*M*, the difference on protein kinase A is mild, but that on polarity is remarkable. In SPs-*C* and SPs-*O*, the majority of the considered physicochemical attributes show a big difference in secreted proteins against the non-secreted proteins.

**Figure 3:**
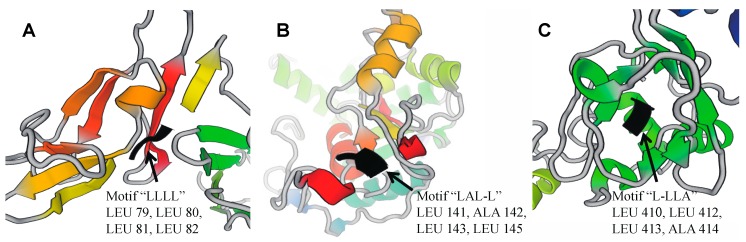
Example of leucine-rich motifs in mammalian secreted proteins. Panels (**A**–**C**) are captured from protein 3D structure 4GRW (human IL-23 with 3 Nanobodies), 1T8T (human 3-*O*-Sulfotransferase-3 with bound PAP), and 5NV6 (human transforming growth factor beta-induced protein), respectively.

Listed in Table 1 are the calculated top 20 informative motifs in various datasets. We find that ‘L’-rich (leucine-rich) MTFs are highly favored in SPs-all and SPs-*H* (exemplified by Figure 3). Extracellular leucine-rich pattern domains are proved to be the key organizers of connectivity among the development of neural circuits in secreted proteins [22]. It also regulates axon guidance, target selection, synapse formation, and the stabilization of connections [23]. The ‘L’-rich MTFs in different secondary structures usually indicates various structure functions. As shown in Figure 3, the ‘L’-rich MTFs are always located at the intrinsically disordered region (Figure 3A, ‘LLLL’ motif), the middle of the coil (Figure 3B, ‘LAL-L’ motif), and the edge of the helix (Figure 3C, ‘L-LLA’ motif). For instance, ‘L’-rich MTFs in α-helices often shows pronounced curvature, while the β-strand usually expresses effective binding interaction [24]. Since ‘L’-rich MTFs is an efficient structure, it endows them the capability of regulating intercellular communication and cell adhesion. This can explain why they are most favored in secreted proteins [24]. ‘G’-rich motifs are prevalent in SPs-*M*, SPs-*C*, and SPs-*O*. These phenomena keep consistent with that in amino acid compositions. However, although ‘C’ is under-represented in secreted proteins, it plays important roles in the compositions of MTFs. The enriched conditions of ‘L’ and ‘G’ might be a reason for such phenomenon. Although ‘C’ residues are depleted in secreted proteins, we find that the ‘C’-rich motifs are enriched in various species of secreted proteins. More detailed information of these MTFs is provided in Table S1. The physicochemical index data for twenty standard amino acids is listed in Table S2.

### 2.2. The Performance of the Extracted Features

In Section 2.1, we analyze the differences across various species of secreted proteins and non-secreted proteins on considered features. However, it is still unknown whether these features can be used to distinguish secreted proteins from non-secreted proteins. Here, we test these features on general SPs-all and five species-specific datasets.

Table 2 shows the prediction performance of the considered different features on the training datasets over five-fold cross-validation. Overall, the features of AAC, MTF, and PCP produce promising results on the general mammalian secreted proteins datasets and six species-specific secreted proteins. In detail, AAC-based features perform the best among three types of features with the highest Matthews Correlation Coefficient (MCC) and AUC values on SPs-all. Although MTF-based features could not achieve the highest prediction performance, they are featured by the high capability in recognizing non-secreted proteins (Specificity > 0.84). For Mammalia, *B. taurus*, and *C. lupus familiaris* secreted proteins, the MTF-based features give out high specificity, which is above 0.9. In comparison with ACC- and MTF-based features, PCP-based features produce similar results on six training datasets.

### 2.3. The Performance of Feature Selection Scheme

We empirically prove the prediction capability of proposed features in Section 2.2. In this section, we combine three types of features together to construct the feature space. When considering the existence of redundant features, we firstly use Fisher-Markov Selector [25] to calculate the coefficients between each of the features and labels. The ranked feature lists are provided in Figure S1. Next, we iteratively add features into the feature subset according to the incremental feature selection strategy.

Table 3 shows the prediction results that are based on the optimal feature subsets. The numbers for six optimum feature subsets are 30, 20, 45, 30, 30, and 35, respectively. They achieve MCC ranging from 0.490~0.644 and AUC ranging from 0.783~0.835. On SPs-*M* datasets, the MCC and AUC increase by 0.061 and 0.033 when compared with the best individual features. On SPs-*B*, the AUC slightly climbs to 0.815. More significant improvements are shown on SPs-*O* (MCC 0.644 versus 0.477) and SPs-*C* (MCC 0.546 versus 0.410). These results illustrate the effectiveness of our feature selection scheme. The prediction performances of the detailed different numbers of features on six training sets are provided in Table S3.

### 2.4. Comparison of Species-Specific Models with Traditional Universal Ones

**Figure 4:**
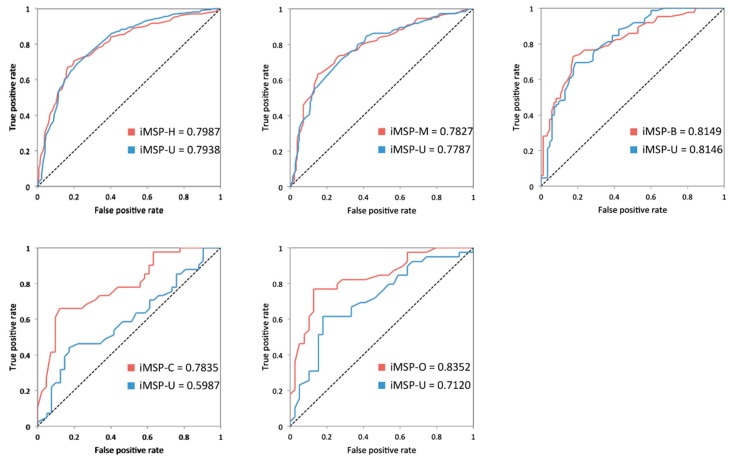
Comparison of predicted AUC values between species-specific and universal models.

Based on our previous investigation, different species of secreted proteins show various attributes in many aspects. Then, we are inspired to introduce species-specific strategy for the specific identification of various mammalian secreted proteins. When compared with universal models, species-specific ones are based on specific feature construction and optimal feature subsets. To investigate the effectiveness of this strategy, we compare these two kinds of models based on same benchmark training datasets over five-fold cross-validation. As shown in Table 4, species-specific models all achieve relatively higher (2~11%) prediction accuracy. The improvements are much more obvious on the sensitivity (3~18%) for different species expect for *M. musculus*. When considering MCC, which is capable of balancing the measurements between sensitivity and specificity, the species-specific model all produce higher values. Figure 4 displays the AUCs of species-specific and universal models. The grey bars indicate the species-specific models, while the black ones stand for the universals. For *H. sapiens*, *M. musculus*, and *B. taurus*, the improvements on AUC are about 0.018, 0.021, and 0.023. For SPs-*C* and SPs-*O*, the AUC values sharply increase from ~0.59 to ~0.78 and ~0.71 to ~0.83.

### 2.5. Comparison with Other Predictors on Independent Testing Datasets

To evaluate the generalization capability of the proposed predictor as well as to compare with previous methods, we further test our method on the independent testing dataset. Recent years have witnessed several powerful predictors for identification of SPs, such as SecretomeP, NClassG+, and SRTpred. The criteria that used for selecting efficient methods include (1) the outputs of the predictors are scores and (2) the predictors can successfully predict an average length protein sequence with 200 residues within 30 min. As a result, we select two predictors, namely SecretomeP [26] and SRTpred [27], as of January 2018.

Table 5 lists the prediction results of considered predictors on various types of testing datasets. The predicted values of SecretomeP and SRTpred are directly obtained through their software. All of the predictors achieve good performance on the universal and various species-specific datasets. Our universal module (iMSP-*U*) produces the MCC of 0.427, 0.455, 0.507, 0.359, 0.324, and 0.332 on six testing datasets respectively. On the former four testing datasets, our iMSP-*U* outperforms SecretomeP and SRTpred. On SPs-*C* and SPs-*O*’s testing sets, SecretomeP and SRTpred show much better than our iMSP-*U*. When adopting species-specific models (iMSP-*H*, iMSP-*M*, iMSP-*B*, iMSP-*C*, and iMSP-*O*) on the corresponding species-specific testing datasets, the prediction performance shows obvious improvements.

### 2.6. Application to Predict Secreted Proteins from Human Proteome by Using iMSP

We implement the proposed method as a public web server, named iMSP, which is deployed at http://www.inforstation.com/webservers/iMSP/. iMSP offers efficient high-throughput predictions for biologists. In this work, our new-compiled benchmark dataset was generated from UniProt (http://www.uniprot.org/, accessed on 1 January 2018). In the UniProt database, sequence similarity search programs are used to identify orthologs. Since *H. sapiens* and *M. musculus* secreted proteins occupy a large part of all secreted proteins, they would somehow influence other species of secreted proteins, such as *B. taurus*, *C. lupus* familiaris, and *O. cuniculus*. In our benchmark dataset, the number of secreted proteins in SPs-*H* and SPs-*M* is much higher than that of SPs-*B*, SPs-*C*, and SPs-*O*. As a result, the accuracy of the latter three species-specific models will be affected by that of the first two. The users are suggested to choose universal model for Bos, Canis and Oryctolagus proteins, and species-specific models for Homo and Mus proteins.

In this part, we aim to adopt iMSP to predict potential secreted proteins from human proteome. There are a total of 71,772 proteins in the human proteome. Among them, 20,303 items are reviewed records, and the rest 51,469 are unreviewed ones. Particularly, by our universal model (iMSP-*U*) and species-specific model (iMSP-*H*), we also calculate the probabilities of unreviewed human proteins to be secreted proteins. All of the proteins were ranked according to the predicted probabilities. Based on iMSP-*H*, we find that 7601 (14.77%) proteins have the probabilities higher than 0.8, while a large number of proteins are not secreted proteins (shown in Table 6). When considering the highest probabilities (≥99%), we find 272 (or 0.528%) out of all 51,469 proteins to be predicted secreted proteins. Finally, we listed the predicted scores for all unreviewed human proteome (Table S4) and potential SPs with highest probabilities (Table S5).

## 3. Materials and Methods

### 3.1. Datasets Preparation

In this study, we collect 17,209 mammalian secreted proteins and 29,479 non-secreted proteins from UniProt. We take consideration of the prevalent several species, which include *Homo sapiens* (*H. sapiens*), *Mus musculus* (*M. musculus*), *Bos taurus* (*B. taurus*), *Canis lupus familiaris* (*C. lupus familiaris*), and *Oryctolagus cuniculus* (*O. cuniculus*). The species-specific datasets are used to explore the differences across the various mammalian secreted proteins. Next, Blastclust [28] is used to cluster these proteins with a threshold of 30%. We pick the longest protein from each cluster as the representative. For each dataset, we randomly pick four-fifths of secreted proteins and an equal number of non-secreted proteins to build the training/cross-validation dataset. The remaining proteins are used for independent testing. Table 7 summarizes the newly-compiled dataset. These datasets are freely available on the iMSP server.

### 3.2. Feature Construction

#### 3.2.1. Amino Acid Composition-Based Features

Amino acids are the fundamental elements of proteins. The features of amino acid composition (AAC) reflect the distribution of amino acids in proteins [29,30]. AAC is widely used in predicting protein function or structures. Given a protein P, the features of AAC are defined, as follows:

(1) $$f_{aa}=\left\{f_{1},f_{2},f_{3},\ldots ,f_{20}\right\}\;$$

where $f_{aa}$ represents the calculated frequency of 20 types of amino acids in the sequence P. Then, these frequencies are normalized to the interval [−1, 1] by using:

(2) $$f_{AAC}=\left(\frac{f_{n}-\min\left(f_{aa}\right)}{\max\left(f_{aa}\right)-\min\left(f_{aa}\right)}-\frac{1}{2}\right)\times 2$$

where $f_{n}$, $\max\left(f_{aa}\right)$, and $\min\left(f_{aa}\right)$ are the original, maximum, and minimum calculated frequency of the amino acids.

#### 3.2.2. Sequence Motif-Based Features

Proteins in the same family tend to share similar attributes. These attributes are usually located on the highly conserved parts of the proteins. These conserved parts can be recognized by sequence patterns/motifs [31]. In this study, we adopt information theory [32] in order to calculate the features of sequence motif (MTF) from protein sequences. Given a protein, the information entropy of the MTF can be formulated, as follows:

(3) I(S)=logN

where N is the number of the considered proteins. Next, we reclassify these proteins with MTF ‘M’. The updated information entropy can be formulated as:

(4) $$I\left(S|M\right)=P\left(M\right)\times \log\left(P\left(M\right)\times N\right)+P\left(\bar{M}\right)\times log\left(P\left(\bar{M}\right)\times N\right)$$

where P(M) represents the percentage of proteins containing ‘M’, while $P\left(\bar{M}\right)$ means the opposite. The Information Gain (IG), which is produced by the introduction of MTF ‘M’ can be calculated as:

(5) IG(M)=I(S)−I(S|M)

In real-world cases, the imbalance on number of SPs to non-SPs would somewhat lead to potential bias on the selected motifs based on IG. Considering this, we further calculate the ratio of the difference value of IG (RDI) for target MTF ‘M’, which is defined as follows:

(6) $$RDI\left(M\right)=\frac{IG_{P}\left(M\right)}{I_{P}\left(S\right)}-\frac{IG_{N}\left(M\right)}{I_{N}\left(S\right)}$$

where $IG_{P}\left(M\right)$ and $IG_{N}\left(M\right)$ are IG of MTF ‘M’ on SPs and non-SPs, $I_{P}\left(S\right)$ and $I_{N}\left(S\right)$ are the original information entropy of secreted proteins and non-secreted proteins. In this study, we select the top 20 informative MTFs to encode each protein. Finally, the feature of MTF is defined as:

(7) $$f_{\mathrm{MTF}}=\left[M_{1},M_{2},\ldots ,M_{20}\right]$$

where $M_{n}$ represents the existence or not of the n-th motif (‘1’ stands for existence; ‘−1’ refers to the opposite).

#### 3.2.3. Physicochemical Properties-Based Features

The physiochemical properties (PCP) of residues reveal microscopic environment of proteins. These microscopic environments includes protein energy, fore, and dynamics [33]. For example, the interfaces are often associated with hydrophobic or polar residues [33]. Graph shape can somewhat determine the surface of the function regions. In view of this, we collect ten popular physicochemical properties to encode the secreted proteins. These properties include hydrophobicity [34], polarity [35], solvation free energy [36], graph shape index [37], transfer of free energy [38], correlation coefficient in regression analysis [39], residue accessible surface area [40], partition coefficient [41], entropy of formulation [42], and protein kinase A [43].

The index data for twenty standard amino acids can be formulated as:

(8) $$\left[\begin{array}{ccc} I_{1,1} & \begin{array}{cc} I_{1,2} & \cdots \end{array} & I_{1,20}\\ I_{2,1} & \begin{array}{cc} I_{2,2} & \cdots \end{array} & I_{2,20}\\ \begin{array}{c} \vdots \\ I_{10,1} \end{array} & \begin{array}{cc} \begin{array}{c} \vdots \\ I_{10,2} \end{array} & \begin{array}{c} \;\\ \cdots \end{array} \end{array} & \begin{array}{c} \vdots \\ I_{10,20} \end{array} \end{array}\right]$$

where $I_{m,n}$ represented the m-th index data for the n-th type of amino acid. Detailed information of these index data are provided in Supplementary Table S1. Given a protein, its total sequence can be mathematically formulated as $SEQ=\left[A_{1},A_{2},\ldots ,A_{L}\right]$, where L is length of the protein, $A_{n}$ is a 20 × 1 submatrix representing amino acids (digital “1” for the occupation and “0” for the opposite). Then, the feature of physicochemical patterns can be formulated as: $f_{\mathrm{PCP}}=\left[PCP_{1},PCP_{2},\ldots ,PCP_{20}\right]$, where $PCP_{n}$ was the average value of the n-th column in the matrix product of Equation (8) and SEQ. These elements are scaled between −1 and 1 using Equation (2).

### 3.3. Feature Selection Strategy

In information theory, the existence of ‘bad’ (noisy or irrelevant) features will potential destroy the classifier or will lead to overfitting [44]. Therefore, it is necessary to remove the bad features before constructing a powerful model. In this study, we introduce Fisher-Markov Selector (FMS) [25], together with incremental feature selection (IFS) strategy to search the optimal feature subset. It uses Markov random field optimization techniques to identify the most informative features in describing the native labels. Incremental feature selection strategy is adopted to build different feature subset, according to the scored feature lists. For each feature subset, a classifier is built and evaluated. The classifier that achieves the highest prediction performance will be chosen as the final prediction model. The corresponding feature subset will be the optimal feature subset.

### 3.4. Model Construction and Performance Evaluation

In this work, LIBSVM 3.20 [33] is utilized to empirically train and optimized the prediction model. The radial basis function is adopted as the kernel function and grid search is used to search for optimal parameters.

We assess our method using two statistical cross-validation methods, namely five-fold cross-validation and the independent test. A five-fold cross-validation is adopted for evaluating the performance of proposed predictor on the training dataset. First, we randomly divide the training dataset into five parts. In each run, four of them are used to train a classifier and test on the holdout fold. Then, we combine the predictions in all five iterations to compute the following threshold-dependent measurements: accuracy, sensitivity, specificity, and Matthews Correlation Coefficient (MCC). They are defined, as follows:

(9) $$Accuracy=\frac{TP+TN}{TP+FP+TN+FN}$$

(10) $$Sensitivity=\frac{TP}{TP+FN}$$

(11) $$Specificity=\frac{TN}{TN+FP}$$

(12) $$MCC=\frac{TP\times TN-FP\times FN}{\sqrt{\left(TP+FP\right)\left(TN+FN\right)\left(TP+FN\right)\left(TN+FP\right)}}$$

where *TP* is the number of correctly recognized secreted proteins, *TN* is the number of correctly recognized non-secreted proteins, *FP* is the number of incorrectly recognized secreted proteins, and *FN* is the number of incorrectly recognized non-secreted proteins. Since the abovementioned threshold-dependent measurements are sensitive to thresholds, we also adopt AUC (area under Receiver Operating Characteristic (ROC) curve), which has been proved to be a robust assessment criterion for imbalanced testing datasets [34].

## 4. Conclusions

Secreted proteins are widely spread in living organisms and cells. Featured by easily being detected in body fluids, urine, and saliva in clinical, they play important roles in potential biomarkers for disease diagnosis and vaccine production. In this study, we present a novel high-throughput predictor for the identification of mammalian SPs from primary protein sequences. We analyze the differences across various types of secreted proteins and non-secreted proteins by using considered features, including AAC, MTF, and PCP. When compared with the traditional universal model, the introduced species-specific scheme proves to be capable of improving the prediction performance for corresponding species of secreted proteins. Tests on independent testing dataset promise a good generalization capability of our proposed method. We also apply the proposed predictor to predict unreviewed human proteome. We list 272 potential secreted proteins, which are predicted with high confidence (≥99%), for further investigation by biologists.

## Figures and Tables

**Table 1:** The top 20 informative motifs in various datasets. ‘-’ denotes arbitrary 20 amino acids.

SPs-All		SPs-*H*		SPs-*M*		SPs-*B*		SPs-*C*		SPs-*O*	
MTF	RDI	MTF	RDI	MTF	RDI	MTF	RDI	MTF	RDI	MTF	RDI
LLLL	0.035	LLLL	0.039	LLLL	0.037	LLLL	0.045	C-CR	0.053	G-CP	0.064
LL-LLL	0.034	LL-LLL	0.038	LL-LLL	0.035	G-CP	0.035	G-CP	0.047	C-VP	0.057
LLL-LL	0.032	LLL-LL	0.036	C-CP	0.027	CP-G	0.033	CG-C	0.047	GC-P	0.052
CP-G	0.022	LAL-L	0.027	C-QG	0.027	C-PG	0.032	C-AG	0.047	CS-C	0.051
LAL-L	0.022	L-LLA	0.024	CP-G	0.026	C-CL	0.032	KGD	0.046	SC-C	0.051
G-TC	0.021	LL-LA	0.024	C-NG	0.024	L-LLA	0.032	CP-Q	0.043	C-SC	0.049
C-PG	0.020	L-LLG	0.024	G-TC	0.024	S-SC	0.032	CC-P	0.043	C-CR	0.049
LLL-A	0.020	LL-LG	0.024	CQ-G	0.024	CS-S	0.031	GR-C	0.042	SC-P	0.047
LL-LA	0.020	LLL-A	0.023	C-PG	0.024	AC-P	0.031	CG-R	0.041	C-SG	0.046
L-LLA	0.020	LLL-G	0.023	GG-C	0.022	LL-LA	0.030	C-CL	0.040	SG-C	0.046
GT-C	0.019	C-PG	0.022	CA-G	0.022	CA-P	0.030	SC-C	0.040	CG-C	0.046
GS-C	0.018	LLA-L	0.022	C-SC	0.022	LLL-A	0.030	CC-R	0.040	GC-G	0.045
L-LW	0.018	CP-G	0.022	C-GG	0.021	LCL	0.029	CV-P	0.039	CC-P	0.044
ALL-L	0.017	ALL-L	0.021	GE-C	0.021	G-SC	0.029	CA-G	0.039	LLLL	0.044
LLA-L	0.017	L-LAL	0.021	GK-C	0.020	SC-S	0.029	PQG	0.037	KPG	0.044
LL-AL	0.017	LL-AL	0.020	GT-C	0.019	C-SS	0.028	CS-C	0.037	C-PT	0.043
G-RC	0.017	LA-LL	0.020	G-SC	0.019	G-CS	0.028	C-SC	0.037	GDR	0.043
P-CP	0.017	AL-LL	0.020	C-PR	0.019	CG-G	0.027	RGP	0.037	CS-G	0.042
CP-P	0.017	G-TC	0.020	WL-L	0.019	AC-S	0.027	C-PT	0.036	C-GC	0.041
CA-P	0.016	L-LW	0.019	G-RC	0.019	A-CL	0.027	PGQ	0.036	S-SC	0.039

**Table 2:** The prediction performance of different features on six training datasets over five-fold cross-validation.

Dataset	Feature	Sensitivity	Specificity	Accuracy	MCC	AUC
SPs-all	AAC	0.695	0.734	0.714	0.429	0.773
	MTF	0.354	0.910	0.632	0.317	0.660
	PCP	0.707	0.702	0.705	0.410	0.754
SPs-*H*	AAC	0.697	0.719	0.708	0.416	0.736
	MTF	0.469	0.846	0.657	0.340	0.677
	PCP	0.670	0.755	0.712	0.426	0.746
SPs-*M*	AAC	0.685	0.734	0.709	0.419	0.754
	MTF	0.361	0.896	0.628	0.304	0.658
	PCP	0.652	0.722	0.687	0.374	0.732
SPs-*B*	AAC	0.663	0.781	0.722	0.447	0.765
	MTF	0.247	0.988	0.618	0.350	0.682
	PCP	0.401	0.953	0.676	0.424	0.731
SPs-*C*	AAC	0.612	0.791	0.701	0.410	0.762
	MTF	0.418	0.925	0.672	0.398	0.667
	PCP	0.463	0.900	0.682	0.404	0.759
SPs-*O*	AAC	0.677	0.797	0.737	0.477	0.744
	MTF	0.563	0.870	0.716	0.454	0.725
	PCP	0.490	0.807	0.648	0.313	0.693

**Table 3:** The performance of the optimum feature subset general Mammalia secreted proteins and five species-specific secreted proteins over five-fold cross-validation.

Dataset	Sensitivity	Specificity	Accuracy	MCC	AUC
SPs-all	0.705	0.783	0.744	0.490	0.806
SPs-*H*	0.673	0.833	0.753	0.513	0.799
SPs-*M*	0.634	0.847	0.740	0.492	0.783
SPs-*B*	0.728	0.825	0.777	0.556	0.815
SPs-*C*	0.657	0.876	0.766	0.546	0.784
SPs-*O*	0.771	0.870	0.820	0.644	0.835

**Table 4:** Comparison between species-specific and universal schemes on different species of training datasets over five-fold cross-validation.

Dataset	Model	Sensitivity	Specificity	Accuracy	MCC
SPs-*H*	iMSP-*H*	0.673	0.833	0.753	0.513
	iMSP-*U*	0.647	0.820	0.733	0.474
SPs-*M*	iMSP-*M*	0.634	0.847	0.740	0.492
	iMSP-*U*	0.652	0.789	0.721	0.446
SPs-*B*	iMSP-*B*	0.728	0.825	0.777	0.556
	iMSP-*U*	0.695	0.811	0.753	0.509
SPs-*C*	iMSP-*C*	0.657	0.876	0.766	0.546
	iMSP-*U*	0.473	0.841	0.657	0.337
SPs-*O*	iMSP-*O*	0.771	0.870	0.820	0.644
	iMSP-*U*	0.615	0.823	0.719	0.447

**Table 5:** The performance of different methods on six testing datasets.

Dataset	Method	Sensitivity	Specificity	Accuracy	MCC	AUC
SPs-all	SecretomeP	0.611	0.798	0.763	0.355	0.729
	SRTpred	0.652	0.824	0.792	0.419	0.781
	iMSP-U	0.590	0.865	0.814	0.427	0.802
SPs-*H*	SecretomeP	0.632	0.787	0.762	0.340	0.764
	SRTpred	0.678	0.802	0.782	0.392	0.770
	iMSP-H	0.631	0.866	0.829	0.443	0.821
	iMSP-U	0.538	0.908	0.850	0.441	0.817
SPs-*M*	SecretomeP	0.629	0.832	0.731	0.471	0.776
	SRTpred	0.707	0.793	0.751	0.503	0.785
	iMSP-M	0.742	0.776	0.759	0.519	0.809
	iMSP-U	0.703	0.802	0.753	0.507	0.803
SPs-*B*	SecretomeP	0.575	0.861	0.824	0.367	0.768
	SRTpred	0.670	0.857	0.833	0.431	0.787
	iMSP-B	0.547	0.901	0.856	0.411	0.795
	iMSP-U	0.679	0.766	0.755	0.327	0.763
SPs-*C*	SecretomeP	0.549	0.921	0.865	0.470	0.779
	SRTpred	0.686	0.866	0.839	0.478	0.782
	iMSP-C	0.412	0.962	0.880	0.457	0.789
	iMSP-U	0.667	0.670	0.670	0.247	0.718
SPs-*O*	SecretomeP	0.729	0.782	0.775	0.390	0.747
	SRTpred	0.792	0.842	0.835	0.509	0.820
	iMSP-O	0.646	0.913	0.876	0.521	0.841
	iMSP-U	0.521	0.805	0.766	0.264	0.716

**Table 6:** Predicted probabilities to be potential secreted proteins in human proteome.

**Probability**	**0–10%**	**10–20%**	**20–30%**	**30–40%**	**40–50%**
iMSP-*U*	1155 (2.24%)	5984 (11.63%)	7803 (15.16%)	7684 (14.93%)	7100 (13.79%)
iMSP-*H*	1904 (3.70%)	6213 (12.07%)	7333 (14.25%)	7219 (14.03%)	6769 (13.15%)
**Probability**	**50–60%**	**60–70%**	**70–80%**	**80–90%**	**90–100%**
iMSP-*U*	5551 (10.79%)	4745 (9.22%)	4028 (7.83%)	3768 (7.32%)	3651 (7.09%)
iMSP-*H*	5536 (10.76%)	4848 (9.42%)	4046 (7.86%)	3993 (7.76%)	3608 (7.01%)

**Table 7:** A breakdown of newly-compiled datasets used in this work.

Dataset	Species	All Dataset	Training Dataset	Testing Daset
		(numP, numN) *	(numP, numN) *	(numP, numN) *
SPs-all	*Mammalia*	(2560, 4299)	(2048, 2048)	(512, 2251)
SPs-*H*	*Homo sapiens*	(1986, 3714)	(1588, 1588)	(398, 2126)
SPs-*M*	*Mus musculus*	(1144, 1147)	(915, 915)	(229, 232)
SPs-*B*	*Bos taurus*	(529, 1148)	(423, 423)	(106, 725)
SPs-*C*	*Canis lupus familiaris*	(252, 492)	(201, 201)	(51, 291)
SPs-*O*	*Oryctolagus cuniculus*	(240, 490)	(192, 192)	(48, 298)

* numP and numN represent the numbers of secreted proteins and non-secreted proteins respectively.

## Supplementary Materials

The following are available online http://www.mdpi.com/1420-3049/23/6/1448/s1. Table S1: The selected 20 motifs in six datasets. Table S2: Physicochemical index data for twenty standard amino acids. Table S3: Performance of different numbers of features in six training datasets over five-fold cross-validation. Table S4: The predicted scores for all unreviewed human proteome by iMSP. Table S5: The predicted scores for potential SPs with highest probabilities by iMSP. Figure S1 Feature ranking in six training sets.
