# Supplementary material for: High-Throughput Identification of Mammalian Secreted Proteins Using Species-Specific Scheme and Application to Human Proteome
Source: Molecules. 2018 Jun 14;23(6):1448. doi: 10.3390/molecules23061448 (PMC6099666; doi:10.3390/molecules23061448)
Supplement: Supplementary file 1 [file molecules-23-01448-s001.zip › molecules-298033-supplementary/molecules-298033-SI.pdf]

SUPPLEMENTARY FILE FOR

High-throughput identification of mammalian secreted proteins using species-specific scheme and application to human proteome

Jian Zhang [1,*,†], Haiting Chai [2,†], Song Guo [1], Huaping Guo [1], Yanling Li [1]

[1] School of Computer and Information Technology, Xinyang Normal University, Xinyang, 464000, P.R. China; jianzhang@xynu.edu.cn; songguo_xynu@yeah.net; hpguoxynu@sina.com; yanlingli639@163.com
[2] College of Medical, Veterinary and Life Sciences, University of Glasgow, Glasgow, G12 8QQ, United Kingdom; h.chai.1@research.gla.ac.uk
***** Correspondence: jianzhang@xynu.edu.cn; Tel.: +86-376-6390765
† Jian Zhang and Haiting Chai contributed equally to this work.

The PDF file includes:

Table S1 The selected 20 motifs in six datasets.

Table S2 Physicochemical index data for twenty standard amino acids.

Table S3 Performance of different numbers of features in six training datasets over five-fold cross-validation.

Figure S1 Feature ranking in six training sets.

Table S1 The selected 20 motifs in six datasets.

SPs-all, I_P = 11.322, I_N = 12.070				SPs-*H*, I_P = 10.956, I_N = 11.895				SPs-*M*, I_P = 10.160, I_N = 10.164			
MTF	IG_P	IG_N	PDV	MTF	IG_P	IG_N	PDV	MTF	IG_P	IG_N	PDV
LLLL	0.714	0.345	0.035	LLLL	0.747	0.342	0.039	LLLL	0.739	0.365	0.037
LL-LLL	0.564	0.192	0.034	LL-LLL	0.593	0.190	0.038	LL-LLL	0.565	0.210	0.035
LLL-LL	0.564	0.214	0.032	LLL-LL	0.592	0.210	0.036	C-CP	0.479	0.206	0.027
CP-G	0.637	0.417	0.022	LAL-L	0.599	0.327	0.027	C-QG	0.534	0.262	0.027
LAL-L	0.567	0.344	0.022	L-LLA	0.627	0.392	0.024	CP-G	0.655	0.394	0.026
G-TC	0.565	0.345	0.021	LL-LA	0.630	0.397	0.024	C-NG	0.526	0.278	0.024
C-PG	0.637	0.433	0.020	L-LLG	0.582	0.348	0.024	G-TC	0.582	0.335	0.024
LLL-A	0.629	0.427	0.020	LL-LG	0.574	0.339	0.024	CQ-G	0.541	0.296	0.024
LL-LA	0.610	0.408	0.020	LLL-A	0.643	0.419	0.023	C-PG	0.661	0.421	0.024
L-LLA	0.609	0.410	0.020	LLL-G	0.568	0.338	0.023	GG-C	0.600	0.371	0.022
GT-C	0.556	0.364	0.019	C-PG	0.645	0.436	0.022	CA-G	0.584	0.358	0.022
GS-C	0.642	0.462	0.018	LLA-L	0.606	0.399	0.022	C-SC	0.541	0.321	0.022
L-LW	0.628	0.456	0.018	CP-G	0.624	0.420	0.022	C-GG	0.622	0.406	0.021
ALL-L	0.588	0.418	0.017	ALL-L	0.600	0.394	0.021	GE-C	0.544	0.328	0.021
LLA-L	0.573	0.406	0.017	L-LAL	0.575	0.374	0.021	GK-C	0.563	0.355	0.020
LL-AL	0.582	0.416	0.017	LL-AL	0.601	0.409	0.020	GT-C	0.584	0.387	0.019
G-RC	0.541	0.375	0.017	LA-LL	0.578	0.389	0.020	G-SC	0.640	0.444	0.019
P-CP	0.585	0.422	0.017	AL-LL	0.593	0.408	0.020	C-PR	0.524	0.328	0.019
CP-P	0.609	0.448	0.017	G-TC	0.539	0.351	0.020	WL-L	0.622	0.430	0.019
CA-P	0.489	0.323	0.016	L-LW	0.625	0.450	0.019	G-RC	0.565	0.375	0.019
SPs-*B*, I_P = 9.050, I_N = 10.165				SPs-*C*, I_P = 7.977, I_N = 8.943				SPs-*O*, I_P = 7.907, I_N = 8.937			
MTF	IG_P	IG_N	PDV	MTF	IG_P	IG_N	PDV	MTF	IG_P	IG_N	PDV
LLLL	0.747	0.384	0.045	C-CR	0.592	0.187	0.053	G-CP	0.784	0.316	0.064
G-CP	0.572	0.281	0.035	G-CP	0.719	0.385	0.047	C-VP	0.660	0.237	0.057
CP-G	0.611	0.355	0.033	CG-C	0.602	0.254	0.047	GC-P	0.640	0.255	0.052
C-PG	0.587	0.331	0.032	C-AG	0.711	0.378	0.047	CS-C	0.599	0.217	0.051
C-CL	0.490	0.226	0.032	KGD	0.631	0.298	0.046	SC-C	0.640	0.264	0.051
L-LLA	0.630	0.384	0.032	CP-Q	0.571	0.254	0.043	C-SC	0.599	0.237	0.049
S-SC	0.639	0.397	0.032	CC-P	0.527	0.207	0.043	C-CR	0.555	0.187	0.049
CS-S	0.648	0.412	0.031	GR-C	0.631	0.331	0.042	SC-P	0.696	0.364	0.047
AC-P	0.496	0.243	0.031	CG-R	0.612	0.323	0.041	C-SG	0.687	0.364	0.046
LL-LA	0.648	0.418	0.030	C-CL	0.549	0.254	0.040	SG-C	0.696	0.379	0.046
CA-P	0.496	0.251	0.030	SC-C	0.612	0.331	0.040	CG-C	0.610	0.282	0.046
LLL-A	0.639	0.415	0.030	CC-R	0.527	0.236	0.040	GC-G	0.696	0.386	0.045
LCL	0.587	0.361	0.029	CV-P	0.571	0.290	0.039	CC-P	0.544	0.217	0.044

G-SC	0.643	0.427	0.029	CA-G	0.641	0.370	0.039	LLLL	0.705	0.401	0.044
SC-S	0.639	0.424	0.029	PQG	0.631	0.378	0.037	KPG	0.687	0.386	0.044
C-SS	0.620	0.409	0.028	CS-C	0.560	0.298	0.037	C-PT	0.567	0.255	0.043
G-CS	0.567	0.352	0.028	C-SC	0.581	0.323	0.037	GDR	0.630	0.332	0.043
CG-G	0.643	0.447	0.027	RGP	0.686	0.441	0.037	CS-G	0.640	0.348	0.042
AC-S	0.540	0.335	0.027	C-PT	0.571	0.315	0.036	C-GC	0.544	0.246	0.041
A-CL	0.648	0.456	0.027	PGQ	0.631	0.385	0.036	S-SC	0.678	0.415	0.039

Table S2 Physicochemical index data for twenty standard amino acids.

	Hydrophobicity	Polarity	SFE	GSI	TFE	CCRA	RASA	PC	EOF	pKa
A	-0.41	8.20	0.68	1.28	0.31	0.69	1.15	0.28	1.54	7.00
R	-0.59	10.50	-2.10	2.34	-1.42	0.59	2.25	0.11	3.41	12.48
D	-1.32	13.10	-1.21	1.60	-0.61	0.63	1.50	0.22	1.95	3.65
C	0.18	5.40	0.39	1.77	0.89	0.26	1.35	0.29	2.20	7.00
Q	-0.91	10.50	-0.22	1.56	-0.71	0.53	1.83	0.36	2.36	7.00
E	-1.23	12.40	-0.77	1.56	-0.71	0.67	1.90	0.34	2.23	3.22
H	-0.65	10.50	0.65	2.99	-0.12	0.59	1.95	0.21	2.43	6.00
I	1.26	5.30	1.89	4.19	0.71	0.56	1.75	0.81	2.33	7.00
G	-0.67	9.00	0.00	0.00	0.32	0.67	0.75	0.18	1.28	7.00
N	-0.92	11.60	-0.61	1.60	-0.48	0.49	1.60	0.26	2.08	8.18
L	1.22	4.91	1.90	2.59	0.51	0.54	1.70	1.00	2.32	7.00
K	-0.67	11.30	-0.57	1.89	-1.80	0.41	2.00	0.09	3.00	10.53
M	1.04	5.70	2.41	2.35	0.41	0.33	1.85	0.74	2.03	7.00
F	1.93	5.30	2.29	2.94	0.49	0.58	2.10	2.18	2.05	7.00
P	-0.49	8.10	1.20	2.67	-0.31	0.60	1.45	0.40	1.80	7.00
S	-0.55	9.20	0.01	1.31	-0.13	0.69	1.16	0.14	1.74	7.00
T	-0.28	8.60	0.52	3.03	-0.20	0.71	1.42	0.23	2.06	7.00
W	0.51	5.40	2.60	3.21	0.31	0.63	2.58	5.71	2.37	7.00
Y	1.67	6.20	1.60	2.94	-0.40	0.50	2.34	1.26	2.29	10.07
V	0.91	5.90	1.51	3.67	0.59	0.53	1.57	0.62	2.08	7.00

SFE, GSI, TFE, CCRA, RASA, PC, EOF and pKa are abbreviations for solvation free energy, graph shape index, transfer free energy, correlation coefficient in regression analysis, residue accessible surface area, partition coefficient, entropy of formulation and protein kinase A respectively.

Table S3 Performance of different numbers of features on six training datasets over five-fold cross-validation.

Species	Number	SN	SP	ACC	MCC	AUC	Number	SN	SP	ACC	MCC	AUC
Mammalia	5	0.307	0.922	0.615	0.290	0.670	**30**	**0.705**	**0.783**	**0.744**	**0.490**	**0.806**
	10	0.632	0.810	0.721	0.449	0.781	35	0.702	0.768	0.735	0.471	0.804
	15	0.676	0.785	0.730	0.464	0.793	40	0.651	0.810	0.730	0.467	0.803
	20	0.683	0.793	0.738	0.479	0.803	45	0.683	0.793	0.738	0.479	0.804
	25	0.678	0.798	0.738	0.480	0.802	50	0.635	0.837	0.736	0.483	0.805
H. sapiens	5	0.604	0.811	0.708	0.425	0.747	30	0.645	0.827	0.736	0.480	0.791
	10	0.704	0.742	0.723	0.447	0.773	35	0.688	0.799	0.744	0.491	0.794
	15	0.698	0.739	0.718	0.437	0.771	40	0.689	0.792	0.740	0.483	0.792
	**20**	**0.673**	**0.833**	**0.753**	**0.513**	**0.799**	45	0.682	0.796	0.739	0.481	0.790
	25	0.689	0.818	0.754	0.512	0.799	50	0.695	0.811	0.753	0.509	0.796
M. musculus	5	0.415	0.831	0.623	0.270	0.635	30	0.634	0.831	0.732	0.474	0.776
	10	0.682	0.809	0.745	0.495	0.765	35	0.661	0.809	0.735	0.475	0.777
	15	0.678	0.781	0.730	0.462	0.769	40	0.634	0.842	0.738	0.486	0.781
	20	0.639	0.789	0.714	0.433	0.770	**45**	**0.634**	**0.847**	**0.740**	**0.492**	**0.783**
	25	0.623	0.836	0.730	0.470	0.774	50	0.650	0.831	0.740	0.489	0.781
B. taurus	5	0.530	0.811	0.670	0.355	0.718	**30**	**0.728**	**0.825**	**0.777**	**0.556**	**0.815**
	10	0.681	0.835	0.758	0.522	0.806	35	0.740	0.799	0.770	0.540	0.803
	15	0.671	0.811	0.741	0.487	0.798	40	0.695	0.846	0.771	0.548	0.809
	20	0.775	0.754	0.765	0.530	0.809	45	0.707	0.835	0.771	0.546	0.812
	25	0.719	0.811	0.765	0.532	0.804	50	0.728	0.816	0.772	0.546	0.813
C. lupus familiaris	5	0.537	0.876	0.706	0.439	0.711	**30**	**0.657**	**0.876**	**0.766**	**0.546**	**0.784**
	10	0.562	0.781	0.672	0.352	0.667	35	0.657	0.856	0.756	0.523	0.779
	15	0.682	0.781	0.731	0.465	0.734	40	0.682	0.831	0.756	0.518	0.775
	20	0.468	0.915	0.692	0.428	0.719	45	0.657	0.856	0.756	0.523	0.781
	25	0.368	0.975	0.672	0.432	0.728	50	0.706	0.781	0.744	0.489	0.767
O.	5	0.667	0.693	0.680	0.359	0.702	30	0.745	0.844	0.794	0.591	0.823

cuniculus	10	0.438	0.948	0.693	0.448	0.743	**35**	**0.771**	**0.870**	**0.820**	**0.644**	**0.835**
	15	0.536	0.948	0.742	0.531	0.807	40	0.771	0.844	0.807	0.616	0.832
	20	0.693	0.844	0.768	0.543	0.806	45	0.745	0.870	0.807	0.619	0.833
	25	0.771	0.823	0.797	0.595	0.822	50	0.792	0.823	0.807	0.615	0.831

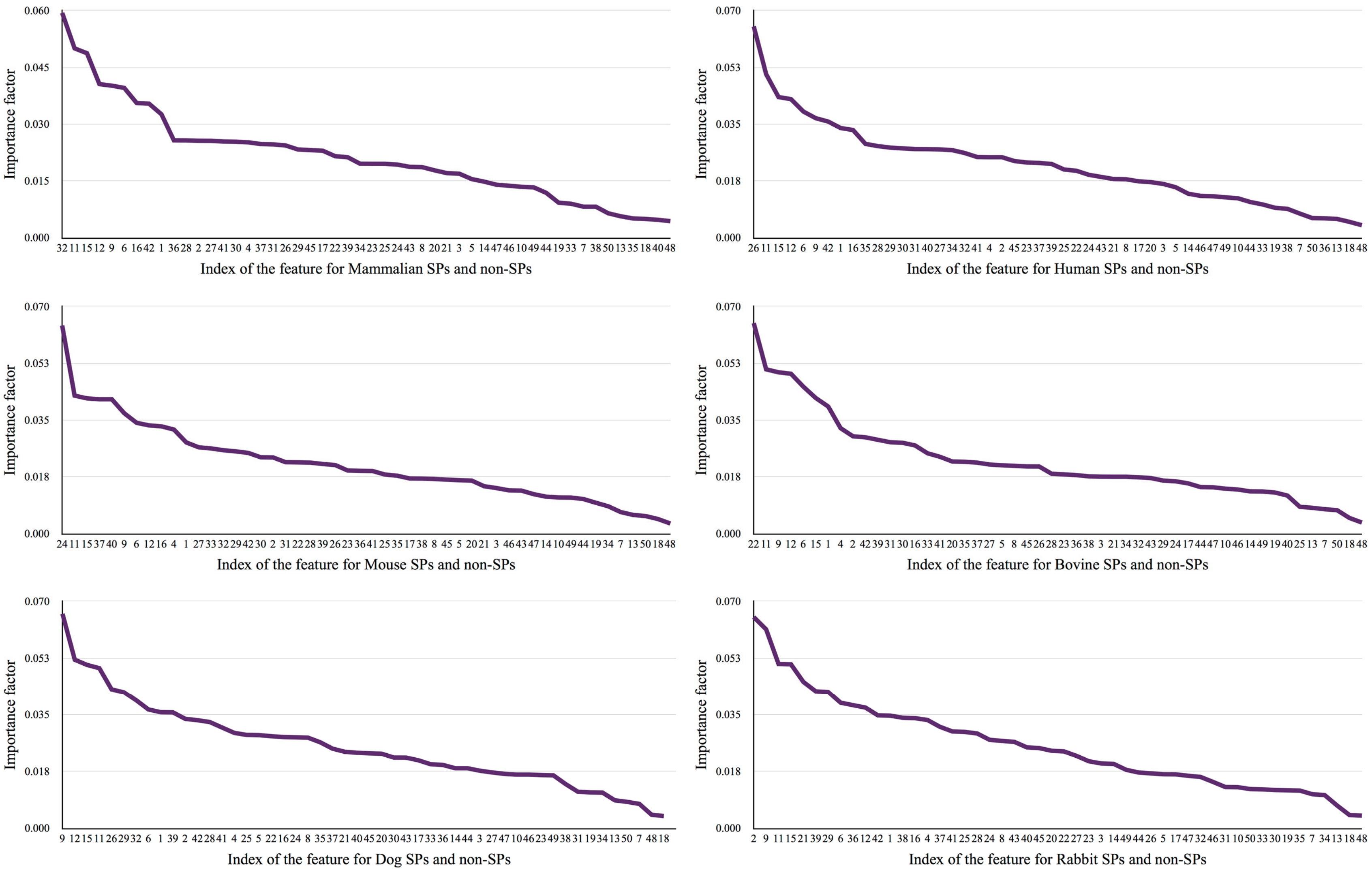

Figure S1 The ranked features according to the correlation coefficients which calculated by Fisher Markov Selector using default parameters.
